# Spatial Transcriptomic Profiling of the Human Placenta in Congenital Cytomegalovirus Infection: A Broad Antiviral Response with Concurrent Suppression of Trophoblast and Vascular Programs

**DOI:** 10.3390/v18101095

**Published:** 2026-10-03

**Authors:** Samantha N. Atkinson, Halli E. Miller, Jason Jarzembowski, Christine Duris, Amy Y. Pan, Li-Chun Lin, Ravit Arav-Boger

**Affiliations:** 1 Center for Microbiome Research, Medical College of Wisconsin, Milwaukee, WI 53226, USA; 2 Department of Pediatrics, Division of Infectious Disease, Medical College of Wisconsin, Milwaukee, WI 53226, USA; 3 Department of Pathology, Children’s Wisconsin, Milwaukee, WI 53226, USA; 4 Department of Pediatrics, Bioinformatics and Quantitative Child Health, Medical College of Wisconsin, Milwaukee, WI 53226, USA; 5 Iowa NeuroBank Core, Iowa Neuroscience Institute, University of Iowa, Iowa City, IA 52246, USA

**Keywords:** congenital cytomegalovirus, placenta, spatial transcriptomics, interferon-stimulated genes (ISGs), trophoblast, maternal–fetal interface, innate immunity

## Abstract

Congenital cytomegalovirus (CMV) is the most common congenital infection and a leading infectious cause of childhood hearing loss and neurodevelopmental disability. The cellular organization of the placental response to infection is incompletely defined. We applied Visium CytAssist spatial transcriptomics to formalin-fixed tissue from two CMV-infected and two uninfected late-gestation placentas and annotated the major maternal and fetal cell populations by reference-based label transfer. Across 16,074 spatial spots, the only significant compositional changes were increased T cell spots and decreased syncytiotrophoblast-enriched spots in CMV-infected placentas; the remaining populations showed similar changes but did not reach significance. Differential expression comparing CMV-infected to uninfected samples within each cell type identified two opposing programs. Infected placentas displayed a broad interferon-stimulated gene signature accompanied by induction of CXCR3-ligand chemokines, antigen-processing machinery, and the major histocompatibility complex class I transactivator *NLRC5*, classical-pathway complement components, and lymphocyte and plasma-cell markers, most pronounced in endothelial, stromal, and syncytiotrophoblast clusters and minimal in extravillous trophoblasts. Concurrently, infected placentas showed downregulation of trophoblast differentiation and syncytialization genes, including *PAPPA2*, pregnancy-specific glycoproteins, *CGA*, and *GCM1*, as well as vascular and hemostatic genes, including *FLT1*, *VEGFA*, *PECAM1*, and *TFPI2*. These data provide a cell-type-resolved molecular correlation of immune activation and placental dysfunction associated with congenital CMV. Together, the data indicate that congenital CMV infection is associated with a coordinated shift at the maternal–fetal interface, in which a broad interferon-driven immune response accompanies suppression of trophoblast-developmental and vascular programs, providing a cell-type-resolved reference for larger studies of placental CMV transmission. Future studies in larger cohorts will be needed to confirm these findings.

## 1. Introduction

Human cytomegalovirus (CMV) is the most common congenital infection worldwide [1,2], and the leading cause of nonhereditary childhood sensorineural hearing loss and developmental delay [1,2,3]. Although maternal immunity to CMV provides partial protection, even in areas such as the United States and Europe, congenital CMV (cCMV) occurs in infants born to seropositive mothers, reflecting reinfection with a new strain or viral reactivation [2,4,5]. In lower-income regions, CMV is acquired universally in early childhood, and seropositivity approaches 100% in women of childbearing age [6,7]. There are no established guidelines for cCMV screening in pregnancy, no approved prenatal interventions to prevent transmission, and no validated prenatal markers of disease [8]. Efforts to develop a CMV vaccine remain ongoing but have been disappointing to date, including the recent Moderna mRNA vaccine (NCT05085366 (ClinicalTrials.gov)) [9,10,11,12,13,14,15]. Understanding the mechanisms that govern placental infection may guide preventive and therapeutic strategies.

CMV transmission occurs via placental infection during maternal viremia, disrupting normal placental development and function [16,17]. The virus infects differentiating cytotrophoblasts and the uterine microvascular endothelium, inducing the production of proinflammatory and angiogenic mediators that impair maternal–fetal vascular exchange [18,19]. Histopathology of cCMV-infected placentas shows increased chronic villitis in symptomatic compared with asymptomatic infants [20], and the consequences for the fetus depend in part on the gestational age at infection [21]. Infection of syncytiotrophoblasts is largely abortive, complicating the interpretation of immune activation in that layer [22].

A central determinant of placental transmission is the innate immune response at the maternal–fetal interface, which acts as an active barrier through pattern recognition receptors such as Toll-like receptors and NOD-like receptors [23,24,25,26,27,28]. We previously reported that multiple CMV strains can cross the placenta [29,30], suggesting that placental immune responses contribute to CMV transmission. However, how the diverse cell populations of the human placenta respond to CMV in situ remains incompletely defined.

Bulk RNA sequencing and in vitro studies cannot resolve which placental cell types mount these responses or how they are spatially organized. Here, we applied spatial transcriptomics to formalin-fixed placental tissue from CMV-infected and uninfected placentas at late gestation to map the cellular response to infection. We identified two concurrent and opposing programs: a broad induction of innate and interferon-driven immunity across resident and infiltrating cell types, and a coordinated suppression of trophoblast developmental and vascular gene programs.

## 2. Materials and Methods

### 2.1. Placental Samples

Five-micron, formalin-fixed, paraffin-embedded placentas were obtained from Children’s Wisconsin and the Johns Hopkins Department of Pathology and classified as CMV-infected or uninfected based on CMV immunohistochemistry, matched for gestational age (Table 1). Late gestation was defined as greater than 24 weeks. The spatial transcriptomics cohort comprised two CMV-infected (placentas 2 and 3) and two uninfected (placentas 1 and 4) late-gestation placentas. The Institutional Review Board determined that the project did not meet the criteria for human-subjects research because the specimens were not individually identifiable and the work did not involve intervention or interaction with living individuals.

### 2.2. Spatial Transcriptomics

**Figure 1:**
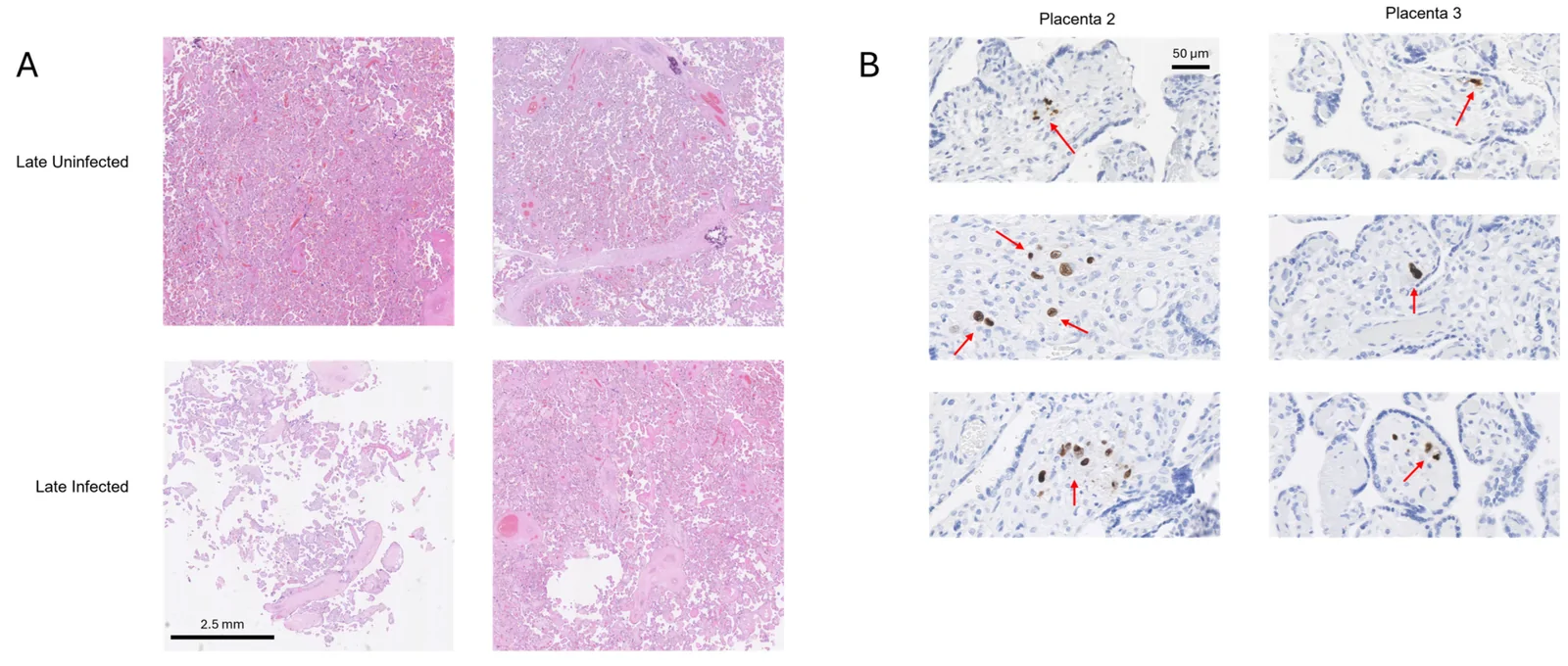
**Tissues used for spatial transcriptomics**. (**A**) Hematoxylin and eosin staining of two uninfected and two CMV-infected late-gestation placentas. (**Top left**) = Placenta 4, (**Top Right**) = Placenta 1, (**Bottom left**) = Placenta 2, (**Bottom right**) = Placenta 3. Scale bars = 2.5 mm at 1× magnification. (**B**) IE1/2 immunostaining confirming CMV infection. Three areas of IE1/2 staining are shown for each infected placenta. Brown (DAB) signal indicates IE1/2 immunostaining, with hematoxylin counterstain. (**Left**) = Placenta 2, (**Right**) = Placenta 3. Scale bars = 50 μm at 20× magnification. Red arrows indicate CMV-positive foci.

Unstained 5 µm sections of formalin-fixed, paraffin-embedded (FFPE) placental tissue from the CMV-infected and uninfected donors were placed on Superfrost Plus slides (Epredia, Kalamazoo, MI, United States) and transferred from the Medical College of Wisconsin to the Iowa NeuroBank Core at the Iowa Neuroscience Institute. Spatial gene expression library preparation was performed according to the Visium CytAssist Spatial Gene Expression protocols for FFPE tissue (10× Genomics; CG000520 and CG000495). After quality assessment of tissue morphology and RNA, one selected section per placenta (four sections in total) was deparaffinized, stained with hematoxylin and eosin, and imaged with a 10× objective on an Olympus VS200 slide scanner (Figure 1A); CMV infection was confirmed by IE1/2 IHC on serial sections (Figure 1B). Sections were decoverslipped, destained, and decrosslinked prior to overnight hybridization with the Human Probe Set v2 (targeting 18,536 genes). The four sections were processed on the Visium CytAssist instrument to transfer analytes onto two Visium CytAssist Spatial Gene Expression slides with 6.5 × 6.5 mm capture areas, followed by probe ligation, release, extension, library construction, and SPRIselect clean-up. Libraries were sequenced on an Illumina NovaSeq 6000 in the Iowa Institute of Human Genetics (IIHG) Genomics Division using the recommended paired-end, dual-indexed configuration (Read 1, 28 cycles; i7 index, 10 cycles; i5 index, 10 cycles; Read 2, 50 cycles). Across the four samples, sequencing yielded a mean of 56,591 to 98,806 read pairs per spot, exceeding the recommended minimum of 25,000 read pairs per tissue-covered spot, with medians of 2728 to 4856 genes and 4440 to 9375 UMIs per spot.

### 2.3. Spatial Data Analysis

Spots were excluded if they were determined not to be under tissue using Loupe Browser v7.0.1 (10× Genomics, Pleasanton, CA, USA), and transcript counts per spot were obtained with Space Ranger v2.1.0 using the human reference probe set (v2.0_GRCh38-2020-A) and default quality-control parameters. We retained 4702, 4924, 1690, and 4758 spots for the two uninfected and two CMV-infected tissues, respectively. The section from Placenta 2 was smaller; therefore, we reported fewer spots for this sample. Individual samples were log-normalized, spots were clustered, and UMAPs were generated; then integration of the four samples (CCAIntegration) was performed, all in Seurat v5.1. [31,32,33,34,35]. CCAIntegration also corrects samples for batch/sequencing bias.

Cell-type identities were assigned by reference-based label transfer (FindTransferAnchors and TransferData, 30 dimensions) using the basal plate with placental villi single-cell RNA-seq dataset of Garcia-Flores et al. [36] as the reference, with reference sub-clusters simplified to primary cell-type categories [37]. Assignments were confirmed against canonical marker gene expression (Supplemental Figure S1). Differentially expressed genes between CMV-infected and uninfected samples within each cell type were identified using FindMarkers (Wilcoxon rank-sum test with Bonferroni correction), with adjusted *p* < 0.05 considered significant. UMAP projections, spatial cell-type maps, spatial feature plots, and the canonical-marker dot plot were generated in Seurat. The pathway heatmaps were generated in Python (v3.12.3) with Matplotlib (v3.10.8) from the average log2 fold changes returned by FindMarkers; each cell shows the log2 fold change for genes with adjusted *p* < 0.05 on a diverging color scale, and non-significant or undetected gene and cell-type combinations are left uncolored. Analyses used R v4.5.1.

### 2.4. Pathway-Level Analysis

To organize differentially expressed genes by biological process, genes were grouped into functional categories: interferon-stimulated and antiviral genes, chemokines, antigen processing and presentation, complement, lymphocyte and plasma-cell markers, trophoblast differentiation and syncytialization, angiogenesis and vascular function, and coagulation and hemostasis. A gene was assigned to a category if it reached significance (adjusted *p* < 0.05) in at least one cell type. The categories were curated by the investigators from published literature on CMV and placental biology and were cross-referenced against the MSigDB Hallmark collection [38] and Gene Ontology Biological Process terms for the placenta-specific, chemokine, and lymphocyte-marker categories [39].

These curated sets are descriptive and were not derived from an unbiased enrichment algorithm. The Results list the specific genes in each category, and Supplemental Table S1 provides complete per-gene, per-cell-type differential expression statistics. Supplemental Table S2 provides log-normalized average expression for every gene by cell type and individual placenta, allowing assessment of whether the described programs were consistent across both infected and both uninfected placentas rather than driven by a single specimen.

### 2.5. Immunohistochemistry for CXCL10 and ISG15

An optimal immunostaining protocol was established using the Leica-Bond Rx (Leica Biosystems, Deer Park, IL, USA) or a Leica Bond MAX automated Immunostaining platform. Formalin-fixed paraffin-embedded tissue sections were cut at 4 μm on a Micron HM355S, placed on poly-L-lysine coated slides and allowed to air-dry until subsequent staining. The antibodies and dilutions using Leica antibody diluent (Leica, Leica Biosystems, Deer Park, IL, USA, AR9352) are as follows: IE1/2 (Millipore, Burlington, MA, USA, MAB810, 1:8000), CXCL10 (Invitrogen, Carlsbad, CA, USA, PA5-79103, 1:200) and ISG15 (Thermo Fisher, Singapore, 15981-1-AB, 1:5000). Following dewaxing (Leica AR9222), antigen retrieval utilized Leica Epitope Retrieval Solution 1 (Leica, AR9661) for 20 min for all antibodies. Primary antibody incubation for ISG15 and CXCL10 was performed for 90 min at room temperature, and 60 min for IE1/2 at room temperature. Immunodetection was performed using the Leica Refine Polymer (Leica, DS9800). Following primary antibody incubations, Leica Refine Kit reagents were incubated as follows with Tris-Buffered saline-Tween (TBST) washing (Leica, AR9590) after each reagent: Post Primary (8 min), Polymer (8 min), peroxidase blocking (5 min), 3,3′ Diaminobenzidine (10 min), and DAB enhancer (5 min) followed by a distilled water wash. All slides were hematoxylin-counterstained and mounted with synthetic mounting media. Omission of the primary antibody served as a negative control. Bright-field images were acquired on a Hamamatsu NanoZoomer 2.0-HT.

## 3. Results

### 3.1. Spatial Transcriptomic Profiling of CMV-Infected and Uninfected Placentas

**Figure 2:**
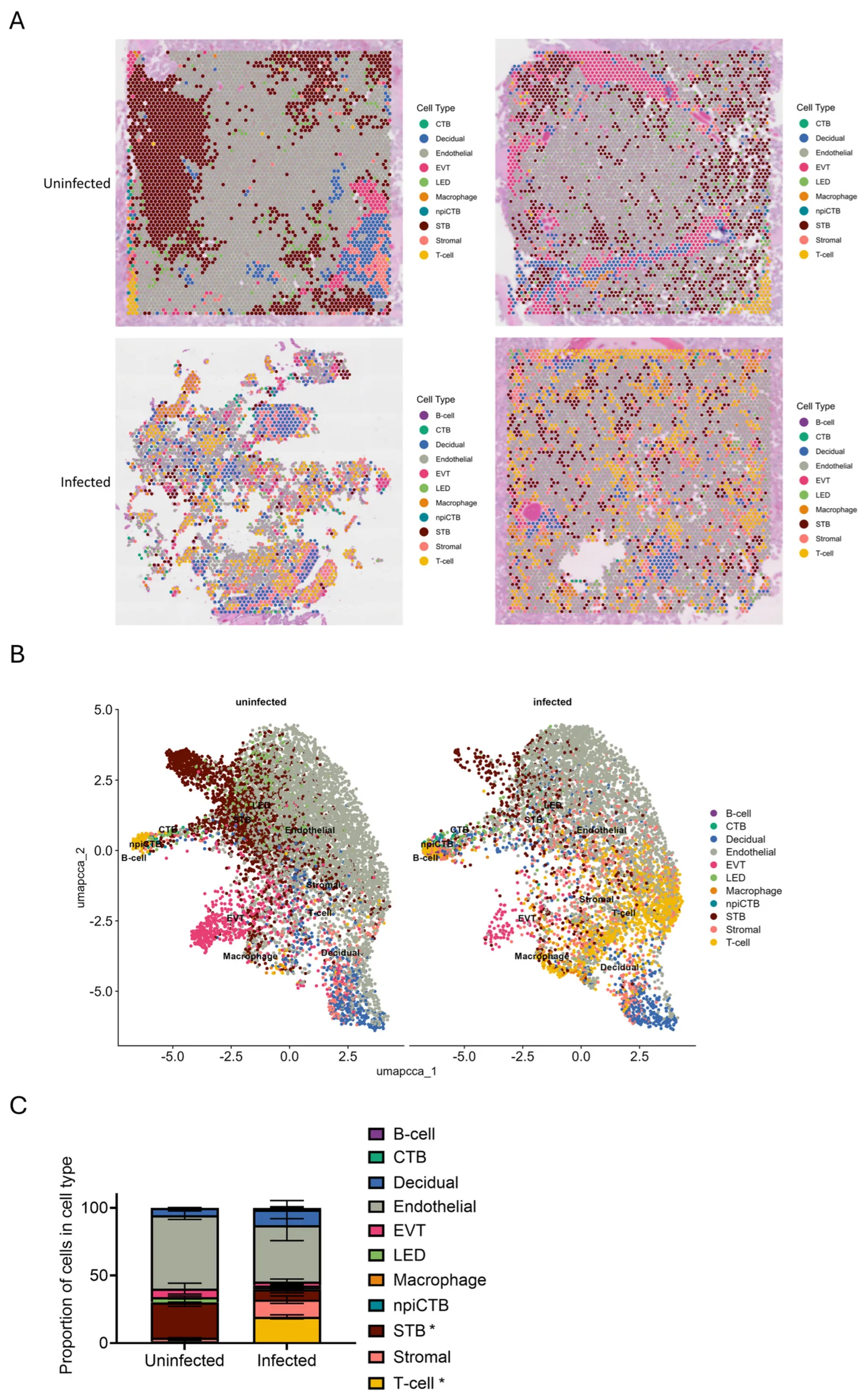
**Spatial transcriptomic profiling and cell-type composition**. (**A**) Spatial plots of annotated cell types mapped onto representative tissue sections in uninfected and CMV-infected placentas. (**B**) UMAP of 16,074 spatial spots from two uninfected and two CMV-infected late-gestation placentas, colored by annotated cluster. Order matches Figure 1A. (**C**) Proportion of spots per cluster by infection status; T cell spots were significantly increased, and STB spots significantly decreased in infection, with non-significant shifts in the other clusters (Supplemental Table S3). EVT, extravillous trophoblast; STB, syncytiotrophoblast; CTB, cytotrophoblast; npiCTB, non-proliferative interstitial cytotrophoblast; LED, lymphoid-endothelial-decidual cells. Two-way ANOVA with Sidak’s multiple comparisons was run on the cell proportions, * *p* < 0.05. Cell type and the interaction term were statistically significant with *p* < 0.000001 and 0.001147, respectively.

We performed unbiased Visium CytAssist spatial gene expression analysis on formalin-fixed, paraffin-embedded tissue from two CMV-infected and two uninfected late-gestation placentas (Table 1). For placenta 2, prenatal ultrasound detected echogenic bowel, and CMV PCR was positive in amniotic fluid. Numerous viral inclusions consistent with CMV were detected in the liver, lungs, kidneys, pancreas, thyroid, and brain. Postmortem liver tissue culture was CMV-positive. For placenta 3, prenatal ultrasound detected microcephaly and hepatosplenomegaly, and eventually intrauterine growth retardation (IUGR), in late gestation. CMV PCR in amniotic fluid was positive. CMV status was confirmed by placental IE1/2 immunostaining and hematoxylin and eosin imaging obtained before capture (Figure 1A,B). After integration of the four samples in Seurat, 16,074 spatial spots were retained. Cell-type identity was assigned by reference-based label transfer using a basal plate and placental villi single-cell reference [36], and verified with a dot plot of canonical marker genes (Figure 2; Supplemental Figure S1). Annotation recovered the major maternal and fetal populations, including syncytiotrophoblast (STB), cytotrophoblast (CTB), extravillous trophoblast (EVT), non-proliferative interstitial cytotrophoblast (npiCTB), villous stroma, decidua, endothelium, a lymphoid-endothelial-decidual (LED) population, macrophages, T cells, and B cells (Figure 2A,B). In the pairwise comparison, STB and T-cells were significant with adjusted *p*-values of 0.005988 and 0.007397, respectively. Spot composition differed between conditions: only the proportion of T cell spots (increased) and STB spots (decreased) reached statistical significance in infected placentas; the remaining clusters showed non-significant changes, with increases in the proportion of B cell, macrophage, CTB, and stromal spots and decreases in the proportion of EVT, npiCTB, and LED spots (Figure 2C; Supplemental Table S3). The decrease in STB spots is consistent with prior reports [40,41]. Differentially expressed genes between CMV-infected and uninfected samples within each cell type (Wilcoxon rank-sum test with Bonferroni correction) are provided in Supplemental Table S1. CMV infection engaged two broad transcriptional programs across placental cell types: induction of innate and interferon-stimulated genes and coordinated suppression of trophoblast and vascular genes, summarized in Table 2. Because significance at the spot level is influenced by the number of spots rather than the number of tested placentas, all comparisons are exploratory.

### 3.2. A Broad Interferon-Stimulated Gene Program Is Induced Across Placental Cell Types

**Figure 3:**
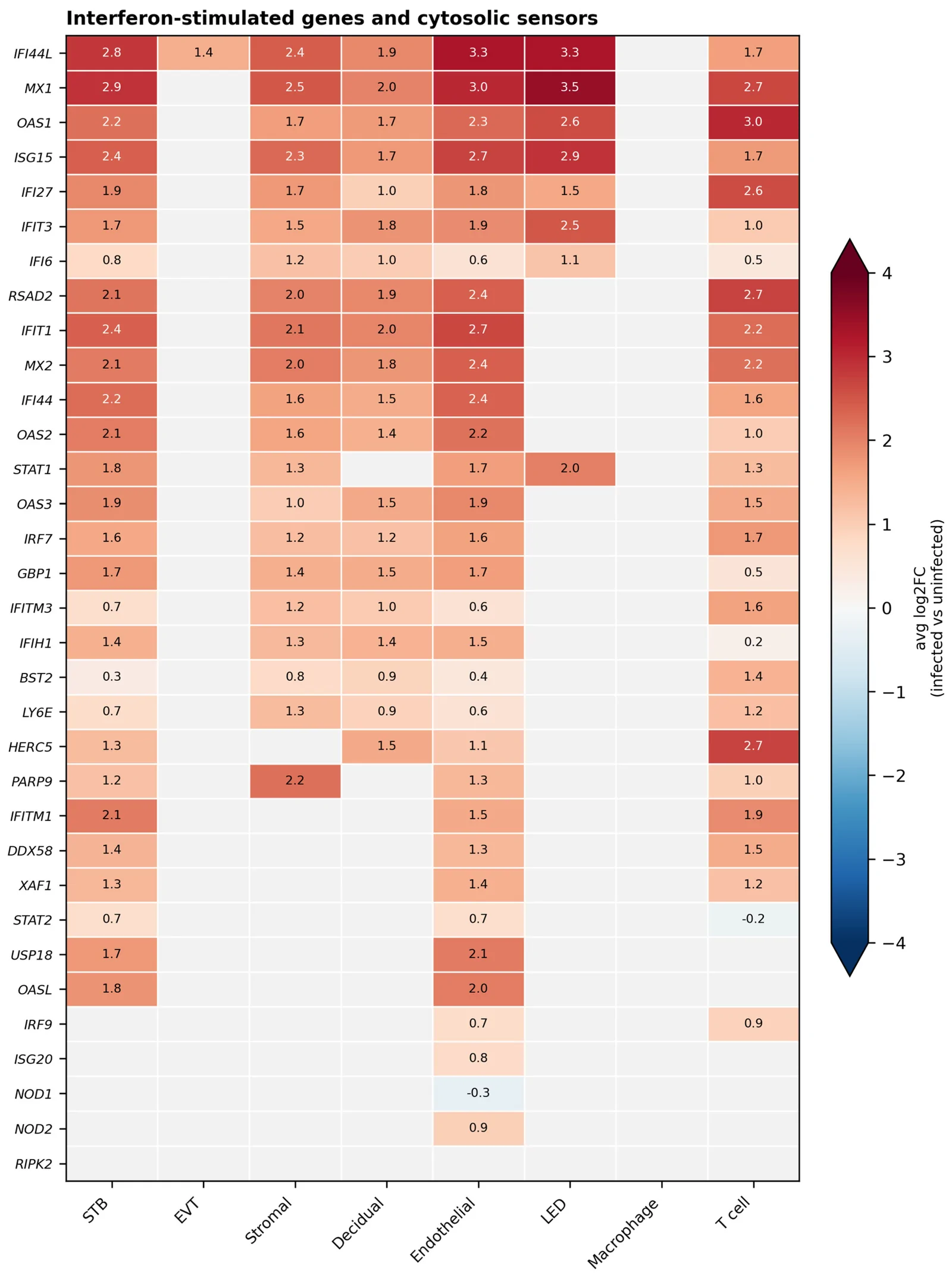
**A broad interferon-stimulated gene program across placental cell types.** Heatmap of representative interferon-stimulated and antiviral genes and the cytosolic and NOD-family sensors across clusters; color encodes the average log2 fold change (infected versus uninfected) for genes with adjusted *p* < 0.05, and grey indicates non-significant or undetected combinations.

Within-cell-type differential expression analysis comparing infected vs. uninfected placentas identified a coordinated interferon-stimulated gene (ISG) program in infected placentas (Figure 3). Induced transcripts included *ISG15*, *MX1*, *OAS1* and *OAS2*, *IFI27*, *IFI44* and *IFI44L*, *IFIT3*, the transcription factors *STAT1* and *IRF7*, and the GTPase *GBP1* (Figure 3). Among cytosolic nucleic-acid sensors, *IFIH1* (*MDA5*) and *DDX58* (*RIG-I*) were induced, whereas the NOD-family sensors *NOD1* and *NOD2* and the adaptor *RIPK2* were not broadly induced. *NOD2* was increased only in the endothelial cluster, *NOD1* was modestly reduced, and *RIPK2* did not change (Figure 3). The program involved the largest number of ISGs in the endothelial and STB clusters, with consistent induction in the T cell, stromal, and decidual clusters and fewer significant ISGs in the LED cluster. EVTs showed minimal ISG induction, and the macrophage cluster, which yielded only 21 significant genes overall, showed no significant ISG induction (Figure 3, Supplemental Table S1). *cGAS* did not reach significance in any cluster, suggesting that ISG induction in this setting engages sensing pathways beyond cGAS-STING [42].

### 3.3. Infection Is Accompanied by Chemokine Induction, Antigen Presentation, Complement Activation, and Lymphocyte Infiltration

**Figure 4:**
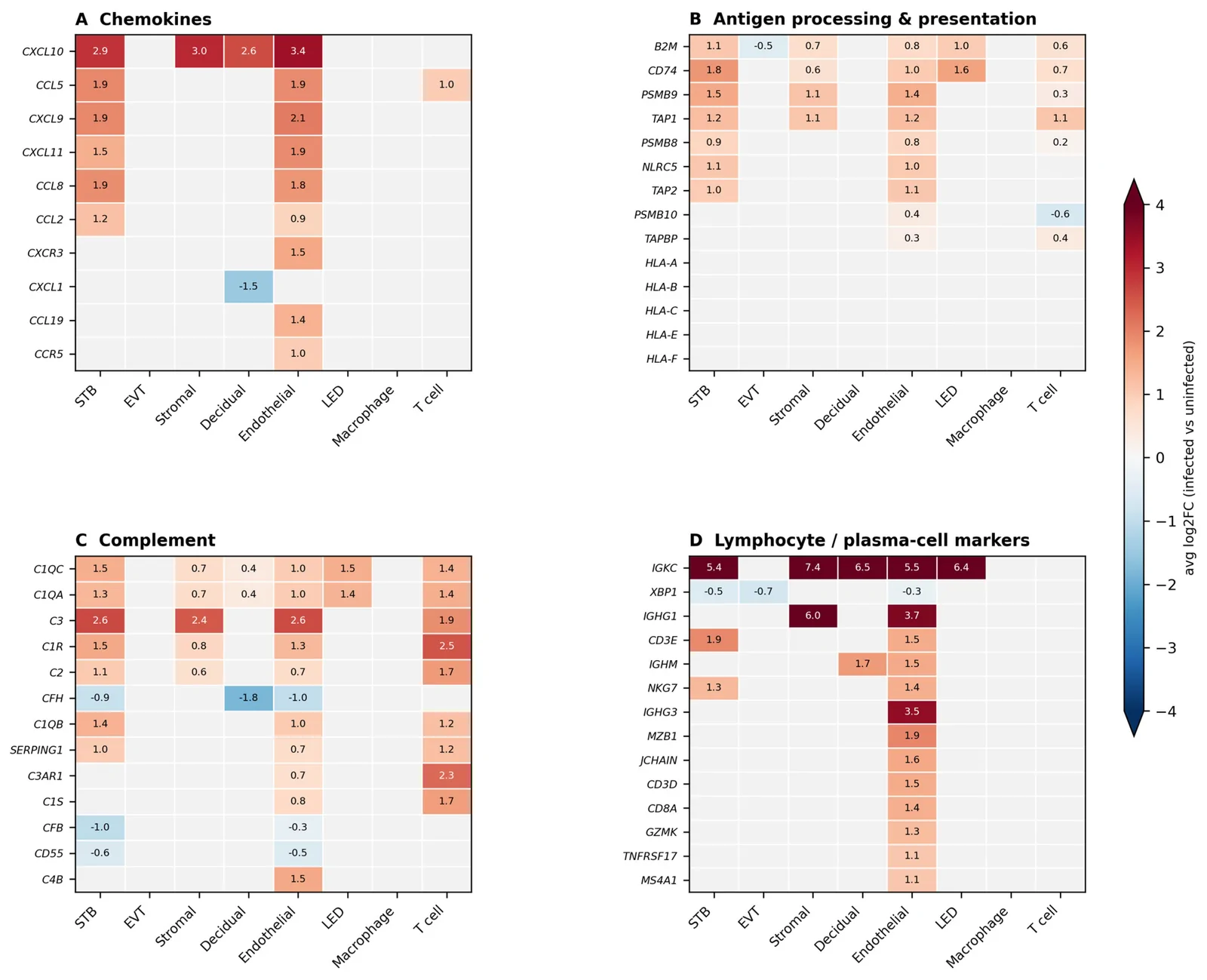
**Chemokine induction, antigen presentation, complement activation, and lymphocyte infiltration**. Heatmaps of differentially expressed (**A**) chemokines, (**B**) antigen-processing and presentation genes, including B2M, TAP1, and NLRC5, (**C**) complement components, and (**D**) lymphocyte and plasma-cell markers, plotted as in Figure 3. Immunoglobulin transcripts reach high fold changes and are color-saturated at the scale maximum.

**Figure 5:**
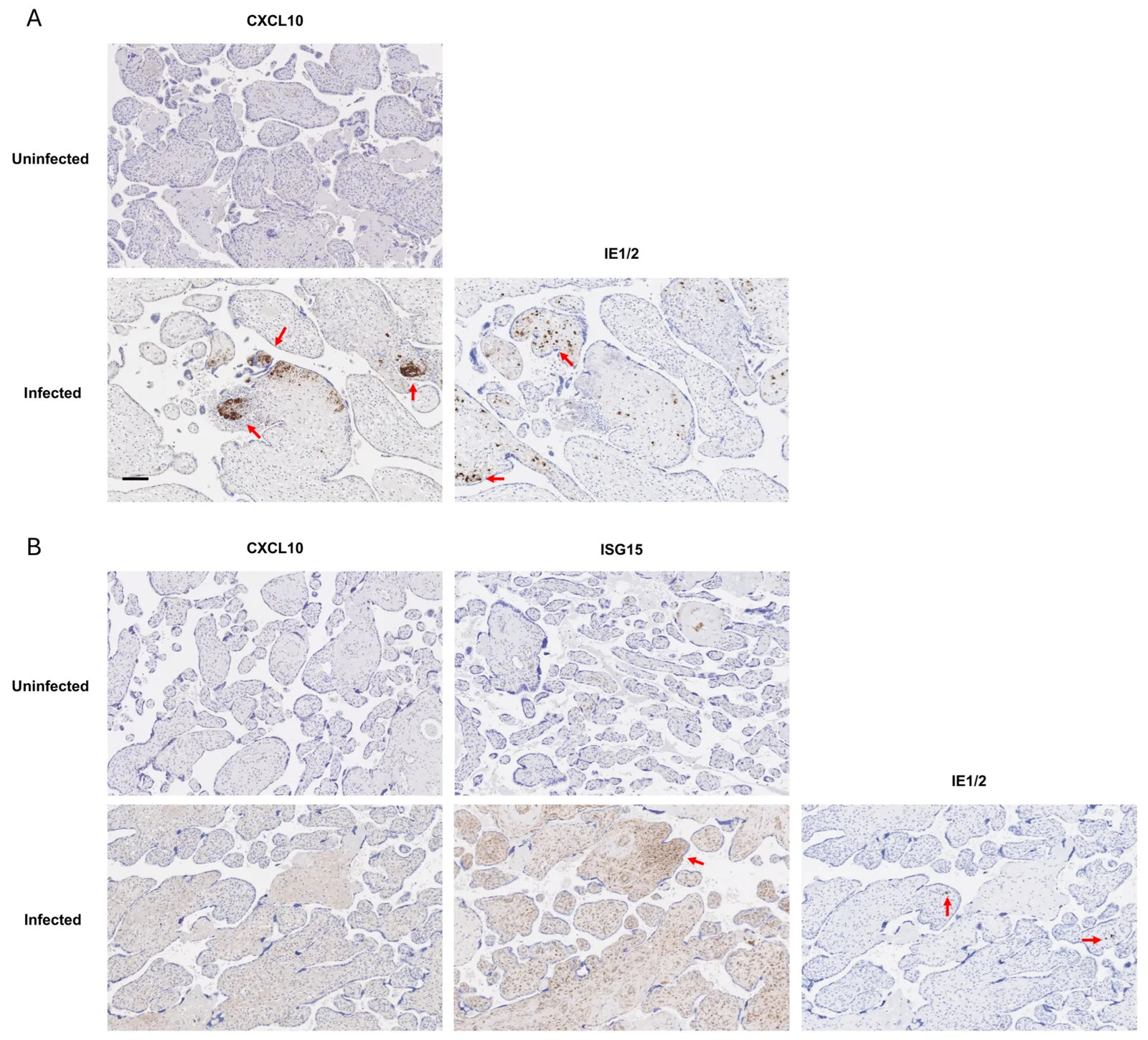
**Immunohistochemistry for CXCL10 (A) and ISG15 (B) in uninfected and CMV-infected placentas.** Brown (DAB) signal indicates the target protein, with hematoxylin counterstain; both proteins show increased expression in infected placentas. Scale bars = 100 µm. Arrowheads indicate representative areas of positive staining. CXCL10 and ISG15 staining were observed predominantly in villous stromal cells. Fields are representative of both infected and uninfected placentas. Because staining was assessed qualitatively without lineage-specific markers, these cells were not definitively identified but may include fibroblasts and macrophages (Hofbauer cells).

The ISG response was notable for the induction of the CXCR3-ligand chemokines *CXCL9*, *CXCL10*, and *CXCL11*, as well as *CCL5* and *CCL8*, most prominently in the endothelial and STB clusters (Figure 4A). *CXCL10* and *ISG15* were chosen as representative, robustly induced markers of the two arms of the response, a CXCR3-ligand chemokine and a core ISG. Spatially, *CXCL10* and *ISG15* transcripts were concentrated in discrete foci in the infected samples (Supplemental Figure S2). For protein-level validation, immunohistochemistry (IHC) confirmed increased expression of CXCL10 and ISG15 in infected placentas (Figure 5). Components of the antigen-processing and presentation machinery were upregulated, including *B2M*, the peptide transporter *TAP1*, the immunoproteasome subunit *PSMB9*, and the class I transactivator *NLRC5*; the HLA class I heavy-chain genes and *HLA-G* were not represented in the Visium probe set and could not be assessed (Figure 4B). Classical complement transcripts increased, including *C1QA*, *C1QB*, *C1QC*, *C1S*, *C3*, and the regulator *SERPING1*, predominantly in the T cell, endothelial, and STB clusters (Figure 4C). Consistent with the compositional increase in immune-cell spots, lymphocyte and plasma-cell signatures were induced, including the immunoglobulin transcripts *IGKC*, *IGHG1*, and *IGHG3* and the plasma-cell markers *MZB1* and *JCHAIN*; because immunoglobulin and hemoglobin transcripts can reflect infiltrating plasma cells and maternal blood, these signals are reported with that caveat (Figure 4D). *NFKBIA*, the principal inhibitor of NF-kB, was reduced in infected STB, LED, endothelial, and decidual clusters (Supplemental Table S1).

### 3.4. Downregulation of Trophoblast Differentiation and Syncytialization Programs

**Figure 6:**
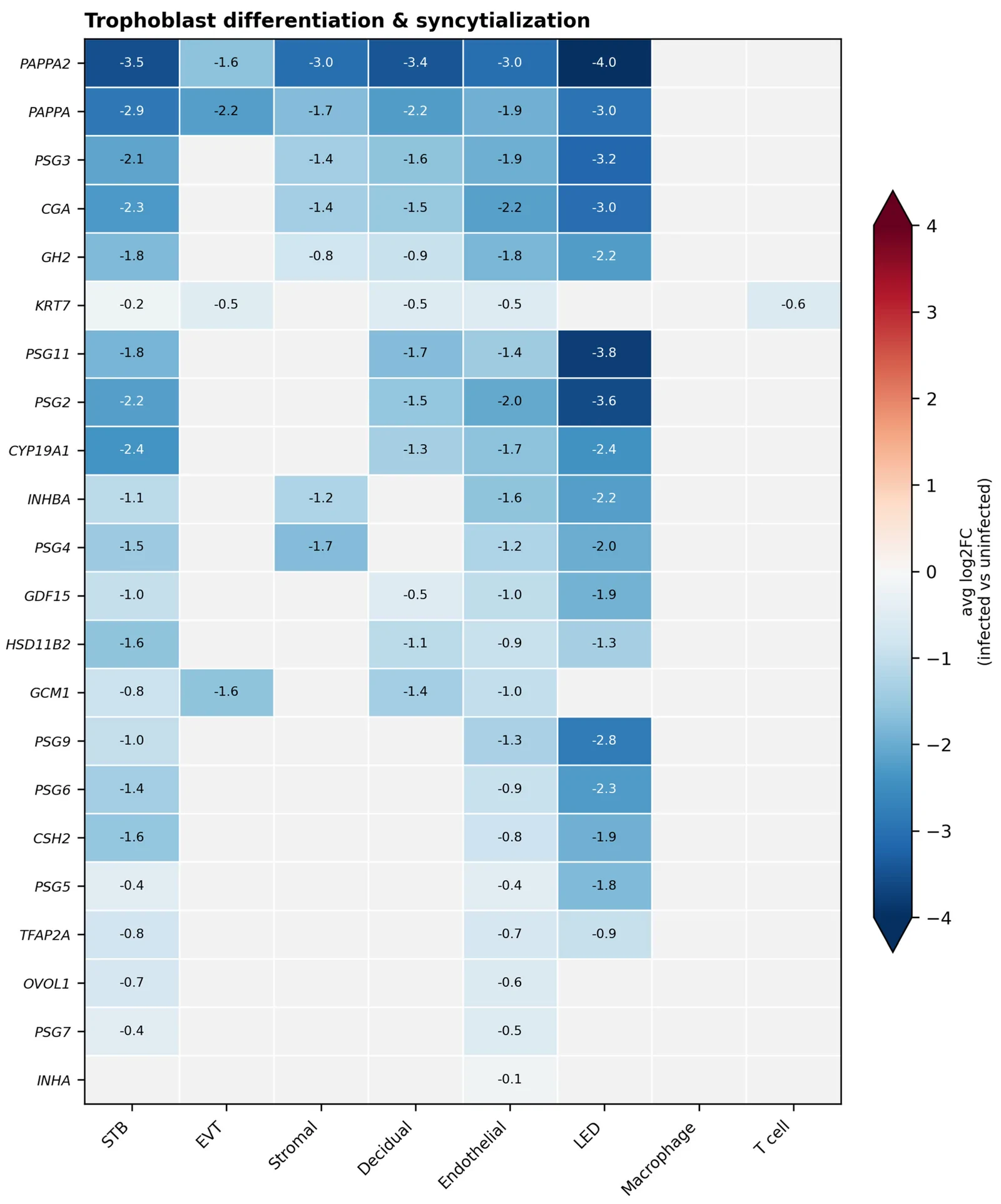
**Downregulation of trophoblast differentiation and syncytialization genes**. Heatmap of trophoblast and placental developmental genes across clusters, plotted as in Figure 3.

In contrast to the induced immune programs, genes associated with trophoblast differentiation and syncytialization were broadly downregulated in infected placentas (Figure 6). These included the pregnancy-associated plasma proteins *PAPPA* and *PAPPA2*, multiple pregnancy-specific glycoproteins such as *PSG3*, the chorionic gonadotropin alpha subunit *CGA*, the steroidogenic enzyme *CYP19A1*, and the master regulator of syncytialization *GCM1*. Downregulation of these genes/programs was evident across the STB, stromal, EVT, LED, endothelial, and decidual clusters, but expression was absent from the T cell and macrophage clusters (Figure 6). This pattern provides a transcriptional correlation of the impaired syncytialization and placental function previously associated with CMV infection [40,41]. *PAPPA2* is of additional interest as a circulating protein linked to adverse pregnancy outcomes.

### 3.5. Vascular and Hemostatic Programs Are Suppressed at the Maternal–Fetal Interface

**Figure 7:**
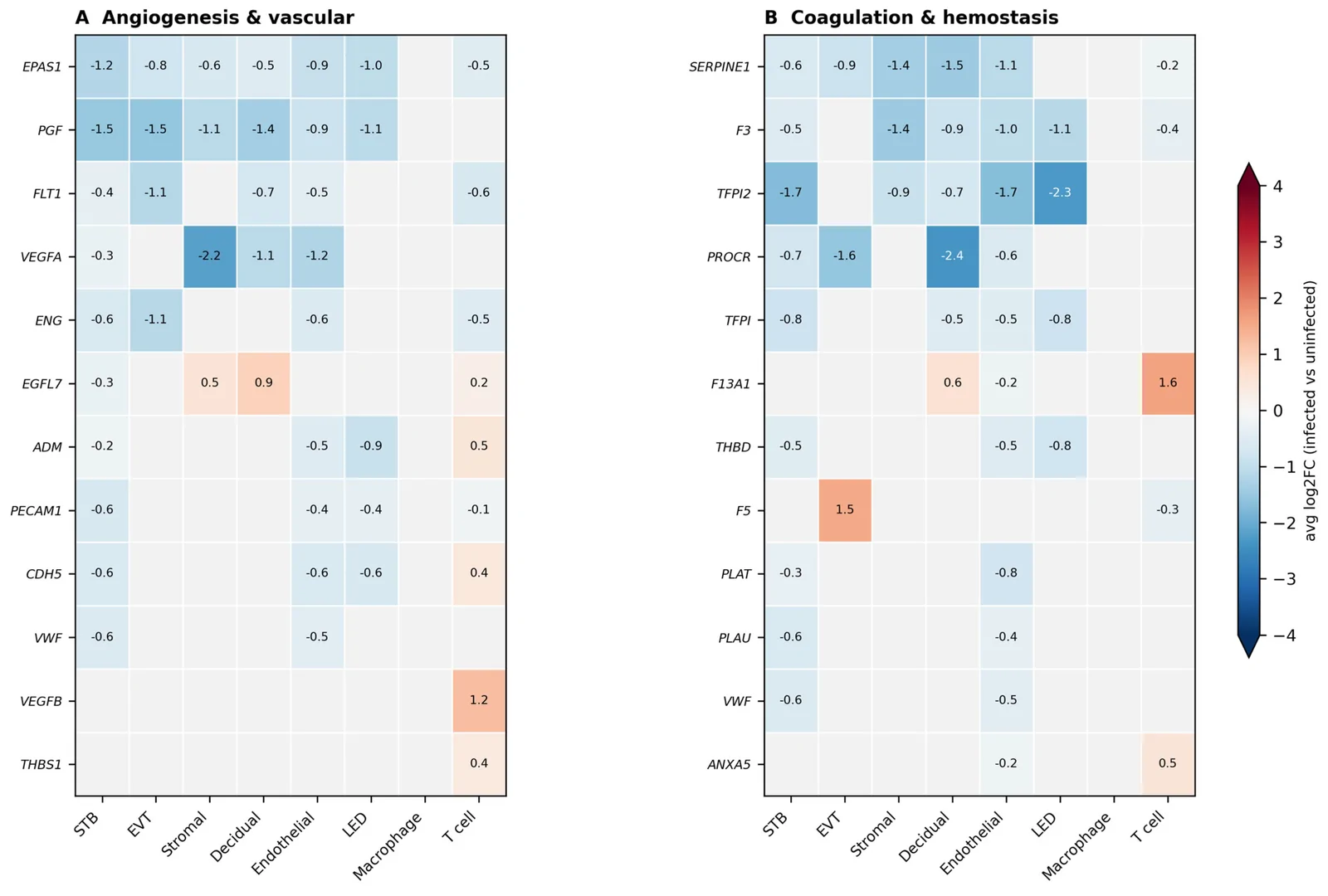
**Suppression of vascular and hemostatic programs.** Heatmaps of differentially expressed (**A**) angiogenesis and vascular genes and (**B**) coagulation and hemostasis genes, plotted as in Figure 3.

Parallel suppression was observed in vascular and hemostatic genes (Figure 7). Angiogenic and endothelial transcripts, including *FLT1, VEGFA, ENG, PECAM1, CDH5*, and the hypoxia-responsive factor *EPAS1*, were reduced, predominantly in the STB, endothelial, LED, and decidual clusters (Figure 7A). Hemostatic and anticoagulant transcripts were also reduced, including the placental factor *TFPI2*, tissue factor *F3*, thrombomodulin *THBD*, the protease inhibitor *SERPINE1*, and the endothelial protein C receptor *PROCR* (Figure 7B).

## 4. Discussion

Spatial transcriptomic profiling of CMV-infected and uninfected placentas identified two concurrent programs at the maternal–fetal interface: broad, cell-type-resolved induction of innate and interferon-driven immunity, and coordinated suppression of trophoblast development and vascular gene programs. Resolving these programs by cell type provides a tissue-level view of the placental response that trophoblast-only systems cannot capture. Because intact placental tissue contains decidual, endothelial, stromal, and immune cells in addition to trophoblasts, this signature represents a composite tissue-level response.

The ISG response, including increased expression of *ISG15* and *MX1*, members of the OAS family, *IRF7*, and the cytosolic sensors *IFIH1* and *DDX58*, is consistent with the type I and type III interferon responses observed in amniotic fluid and decidual tissue during congenital CMV infection [43,44]. The ISG response was broadly distributed and strong in the endothelial and STB clusters, as well as in the stromal, decidual, and T cell clusters, whereas the EVT cluster was largely spared. This contrasts with the trophoblast-intrinsic, DUX4-driven program described in trophoblast organoids [45]; consistent with that distinction, DUX4-stimulated genes were not detected above background in any cluster, in line with the trophoblast-restricted nature of that program and the limited sensitivity of the Visium platform for low-level expressed transcripts in mixed-cell spots. *cGAS* did not reach significance in any cluster, suggesting *ISG15* and related effectors may be induced through sensing pathways beyond cGAS-STING [42]. At the protein level, IHC showed increased staining of ISG15 and CXCL10 in infected placentas (Figure 5), providing qualitative support for their transcriptional induction. Staining for CXCL10 and ISG15 relative to IE1/2 indicated that both infected and neighboring uninfected cells generated an interferon response, in agreement with the transcriptomics data. Consistent with these observations, a recent spatial transcriptomic study of guinea pig CMV placental infection reported a localized immune response driven by chemokine signaling, with prominent induction of CXCL9, CXCL10, and CXCL11 and interferon-responsive pathways [46], suggesting that a chemokine- and interferon-dominated placental response to CMV is conserved across species.

Type III interferon (IFN-lambda) is an important antiviral cytokine at the maternal–fetal interface, with trophoblasts acting as both producers and responders [47,48]. In our dataset, the shared receptor subunit *IL10RB* was broadly expressed across placental cell types, whereas the IFN-lambda-specific subunit *IFNLR1* and the ligands *IFNL1* and *IFNL2* were detected only at low levels (Supplemental Figure S3), indicating limited engagement of the type III interferon axis at the maternal–fetal interface. The interferon-stimulated gene signature observed here is induced by both type I and type III interferons and cannot by itself distinguish between them; given the limited type III engagement, the response is most consistent with type I interferon signaling. Infection was also associated with induction of antigen-processing and presentation machinery, including *B2M*, *TAP1*, the immunoproteasome subunit *PSMB9*, and the MHC class I transactivator *NLRC5*. This is notable at a site that normally restrains classical MHC class I expression to preserve maternal–fetal tolerance [49]. The non-classical molecule HLA-G, which mediates tolerance by inhibiting NK and T cell responses, could not be assessed because it is absent from the Visium Human Transcriptome Probe Set V2 owing to extensive sequence homology across HLA class I paralogs. Whether interferon-driven upregulation of antigen presentation perturbs the balance between tolerance and antiviral defense is an important question that our data cannot resolve and that will require *HLA-G*-specific approaches such as RNAscope or multiplex IHC.

Concurrent with this immune activation, genes governing trophoblast differentiation and syncytialization, including *PAPPA*, *PAPPA2*, pregnancy-specific glycoproteins, *CGA*, *CYP19A1*, and the syncytialization regulator *GCM1*, were broadly downregulated. This provides a transcriptional correlate of the suppression of trophoblast syncytialization reported during CMV infection [40,41]. One plausible link between the two programs is the interferon-induced IFITM proteins, which inhibit cytotrophoblast fusion into STB and can promote fetal demise [50,51]; their induction during infection may contribute to impaired syncytialization. *NFKBIA*, the principal inhibitor of NF-kB, was reduced rather than increased in infected STB, endothelial, decidual, and LED clusters, a pattern compatible with sustained NF-kB activity.

Vascular and hemostatic genes were also suppressed, including *FLT1*, *VEGFA*, *ENG*, *PECAM1*, and the endothelial regulators of coagulation *TFPI2*, *F3*, and *THBD*. Notably, *FLT1* and *ENG* were decreased, in contrast to the elevated soluble FLT1 and endoglin that characterize preeclampsia; this pattern is more consistent with endothelial loss or dysfunction than with the anti-angiogenic secretory state of preeclampsia. These changes are consistent with the dual-edged nature of interferon signaling during pregnancy, although our study does not directly address pathological outcomes [16,48,52].

Transcripts encoding the NOD-family sensors *NOD1* and *NOD2* and the adaptor *RIPK2* were not broadly induced, with *NOD2* increased only in the endothelial cluster. We have previously shown that *NOD2* and its ligand, muramyl dipeptide, restrict CMV replication through an interferon-beta-dependent pathway, and that *NOD1* contributes to the control of CMV in vitro and in vivo [53,54,55]; the absence of broad transcriptional induction of these sensors here does not exclude a functional contribution. Since messenger RNA abundance does not always correlate with protein abundance, post-transcriptional and post-translational mechanisms could regulate protein abundance independently of transcript level [56,57].

Our study has several limitations. The cohort comprised two infected and two uninfected placentas, and statistical significance at the spot level reflects the large number of spots per sample rather than biological replication across individuals; the findings require confirmation in larger, independent cohorts. However, similar patterns of IE1/2 staining were observed qualitatively between the two infected placentas, with sporadic strong staining observed throughout the villi of the placentas, which is completely absent from the uninfected samples. To assess whether the transcriptional programs were consistent across specimens rather than driven by a single placenta, average expression per gene was tabulated by cell type for each individual placenta (Supplemental Table S2), and the interferon-stimulated and trophoblast programs were evident in both infected placentas. The samples span gestational ages from 25 to 40 weeks; therefore, differences attributed to infection could partly reflect gestational age. The pathway summaries are based on curated, hypothesis-driven gene sets rather than unbiased enrichment, and genome-wide differential expression with formal pathway enrichment in a larger cohort is an important future direction. Clinical variables, including maternal serostatus, viral load, and timing of infection, were not available for these archival specimens.

Spatial transcriptomics of the human placenta during cCMV infection reveals a coordinated antiviral and immune recruitment response across resident and infiltrating cell types, occurring alongside suppression of trophoblast developmental and vascular programs. These data provide a cell-type-resolved molecular correlation of the immune activation and placental dysfunction associated with cCMV and serve as a resource for generating testable hypotheses about how antiviral immunity and placental function are balanced at the maternal–fetal interface.

## 5. Conclusions

In late-gestation placentas, congenital CMV infection is accompanied by two coordinated yet opposing transcriptional programs, resolved at the level of individual cell types: a broad induction of interferon-driven and innate immune responses, with immune-cell recruitment, and a concurrent suppression of trophoblast developmental and vascular gene programs. Although based on a small number of placentas, these findings provide a cell-type-resolved molecular correlate of the immune activation and placental dysfunction associated with congenital CMV, and a resource for future studies on how antiviral defense and placental function are balanced at the maternal–fetal interface. Confirmation in larger, independent cohorts and functional studies will be required to establish these patterns and their consequences.

## Figures and Tables

**Table 1:** Placentas analyzed by spatial transcriptomics.

Placenta	Gestational Age (wk)	CMV Status	Confirmation	Platform
1	25	Uninfected	IE1/2 IHC	Visium CytAssist
2	25 to 28	Infected	IE1/2 IHC	Visium CytAssist
3	37	Infected	IE1/2 IHC	Visium CytAssist
4	40	Uninfected	IE1/2 IHC	Visium CytAssist

**Table 2:** Differentially expressed programs in CMV-infected placentas.

Program	Direction	Representative Genes	Clusters with Strongest Signal
Interferon-stimulated/antiviral	Up	*ISG15*, *MX1*, *OAS1*, *IFI44L*, *IFIT3*, *STAT1*, *IRF7*, *IFIH1*, *DDX58*	Endothelial, STB, LED, T cell, stromal
Chemokines	Up	*CXCL9*, *CXCL10*, *CXCL11*, *CCL5*, *CCL8*	Endothelial, STB
Antigen processing and presentation	Up	*B2M*, *TAP1*, *PSMB9*, *NLRC5*	STB, endothelial, stromal
Complement (classical pathway)	Up	*C1QA*, *C1QB*, *C1QC*, *C1S*, *C3*, *SERPING1*	T cell, STB, endothelial
Lymphocyte/plasma-cell markers	Up	*IGKC*, *IGHG1*, *MZB1*, *JCHAIN*, *CD3D*	Endothelial, STB
Trophoblast differentiation/syncytialization	Down	*PAPPA*, *PAPPA2*, *PSG family*, *CGA*, *CYP19A1*, *GCM1*	STB, LED, endothelial, decidual
Angiogenesis/vascular	Down	*FLT1*, *VEGFA*, *ENG*, *PECAM1*, *CDH5*, *EPAS1*	STB, endothelial, LED, decidual
Coagulation/hemostasis	Down	*TFPI2*, *F3*, *THBD*, *SERPINE1*, *PROCR*	STB, endothelial, stromal, decidual

## Data Availability

The spatial transcriptomics data generated in this study are available in the Gene Expression Omnibus (GEO) under accession number GSE329659. Analysis scripts are available at the GitHub repository: https://github.com/MCW-CMR/GSE329659_scripts (Generated on 1 May 2026).

## Supplementary Materials

The following supporting information can be downloaded at: https://www.mdpi.com/article/10.3390/v18101095/s1, Supplemental Figure S1: Dot plot of selected marker genes across cell clusters to verify predicted cell type annotations. Dot plot of canonical genes that define cell types to verify predicted cell type assignments. Expression of *CGA*, *PAPPA2*, *CD68*, *CD4,* and *VWF* was examined across cell types to confirm the accuracy of our predicted cell-type assignments. These genes are canonical markers for STB (*CGA*), EVT (*PAPPA2*), macrophages (*CD68*), T-cells (*CD4*), and endothelial cells (*VWF*). EVT, extravillous trophoblasts; STB, syncytiotrophoblasts; CTB, cytotrophoblasts; npiCTB, non-proliferative interstitial cytotrophoblasts; LED, lymphoid endothelial decidual cells. Supplemental Figure S2: Spatial plots showing the level of expression of *CXCL10* and *ISG15* in CMV-infected and uninfected samples. (A) Expression of *CXCL10* in uninfected (top) and CMV-infected (bottom) placentas. (B) Expression of *ISG15* in uninfected (top) and CMV-infected (bottom) placentas. The warmer the color, the higher the LOG_2_ expression of the gene. Supplemental Figure S3: Mean log-normalized expression of IFN-lambda pathway genes and IL-1 receptor genes across placental cell clusters in uninfected (U) and CMV-infected (I) samples. Color indicates mean log-normalized expression. B cell spots were absent from uninfected samples (grey). Supplemental Table S1: Differentially expressed genes in each predicted cell type cluster comparing infected placentas to uninfected placentas. Supplemental Table S2: Normalized Log2 counts of genes stratified by cell type and individual placenta. Supplemental Table S3: Number of spots in each predicted cell type cluster for uninfected and infected placentas.
