# Supplementary material for: Spatial Transcriptomic Profiling of the Human Placenta in Congenital Cytomegalovirus Infection: A Broad Antiviral Response with Concurrent Suppression of Trophoblast and Vascular Programs
Source: Viruses. 2026 Oct 3;18(10):1095. doi: 10.3390/v18101095 (PMC13709714; doi:10.3390/v18101095)

Supplemental Figure 1:

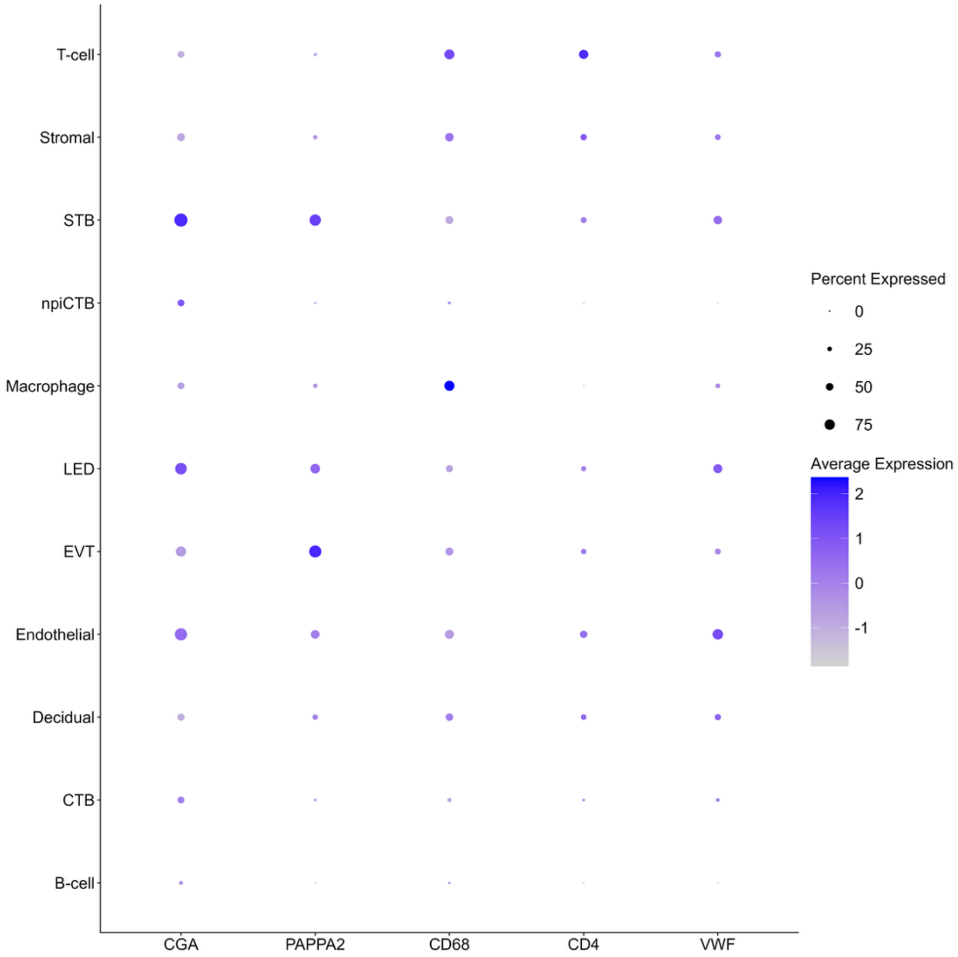

Supplemental Figure 2:

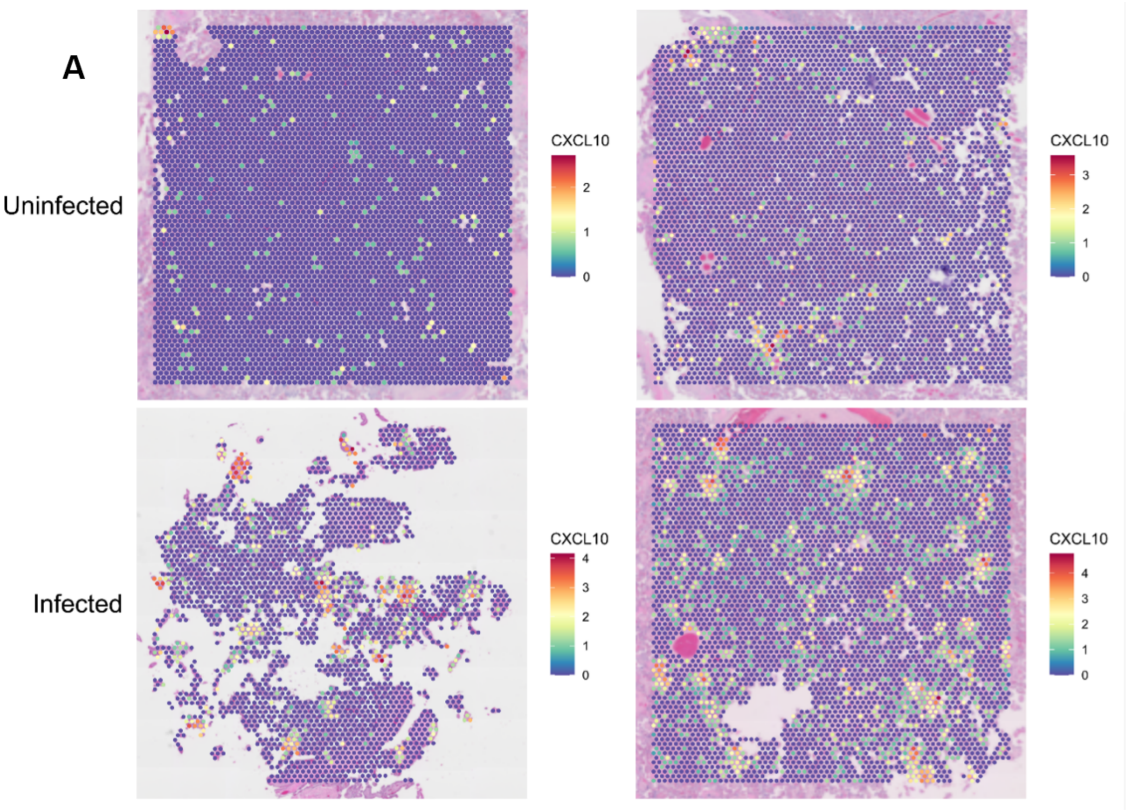

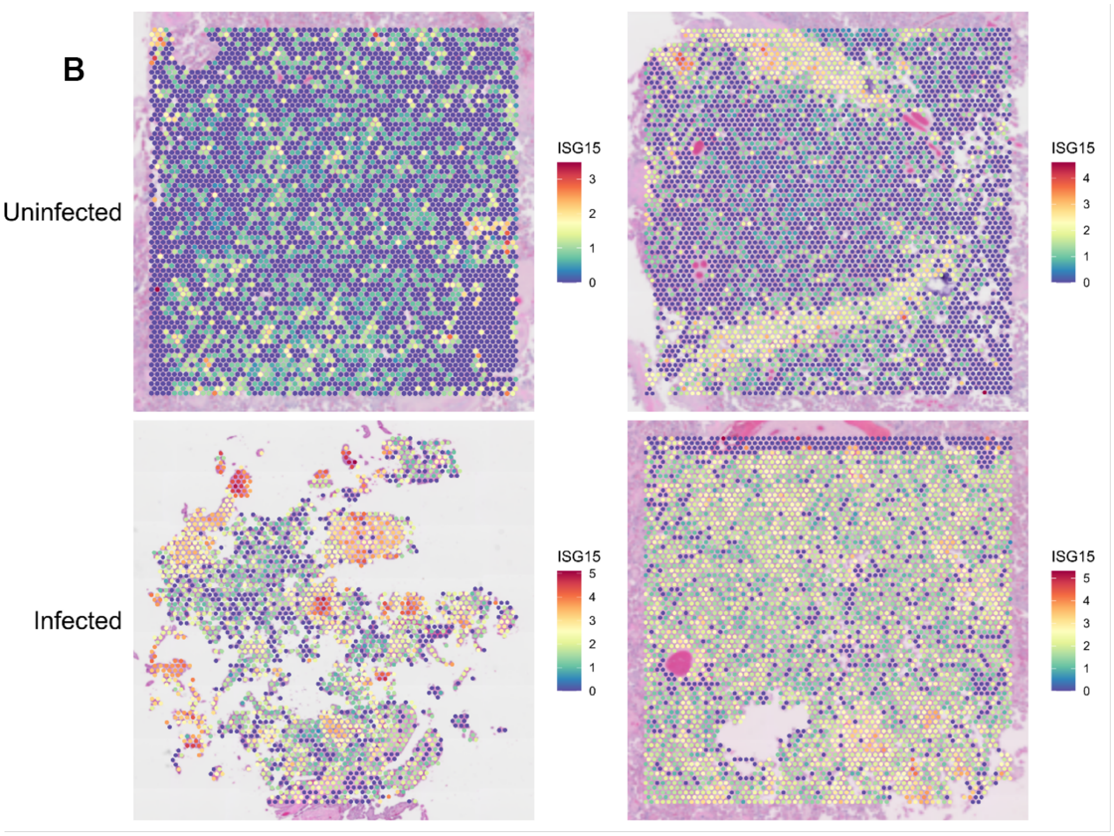

Supplemental Figure 3:

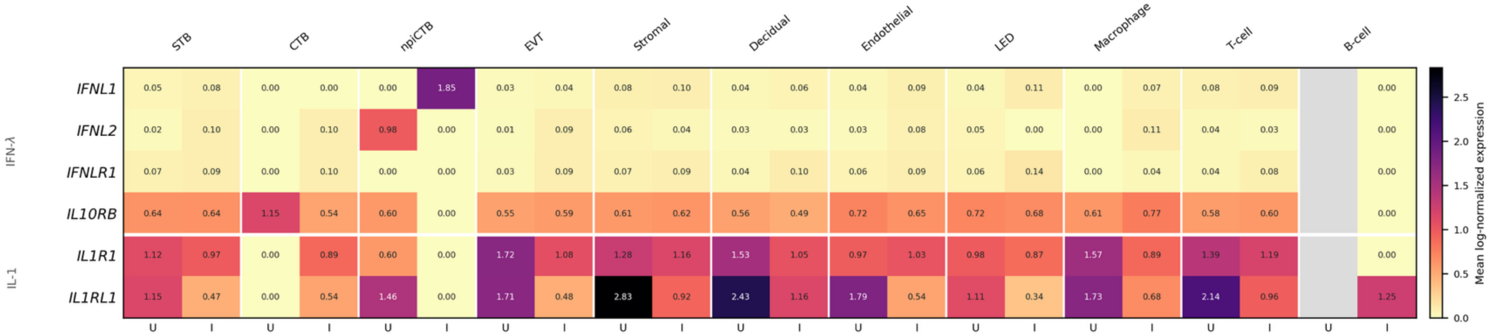

Supplement: Supplementary file 1 [file viruses-18-01095-s001.zip › Suppl Figures _submission.pdf]
