# Supplementary material for: Spatial Transcriptomic Profiling of the Human Placenta in Congenital Cytomegalovirus Infection: A Broad Antiviral Response with Concurrent Suppression of Trophoblast and Vascular Programs
Source: Viruses. 2026 Oct 3;18(10):1095. doi: 10.3390/v18101095 (PMC13709714; doi:10.3390/v18101095)
Supplement: Supplementary file 1 [file viruses-18-01095-s001.zip › Suppl Table 1 for submission.pdf]

Suppl Table 1

	uninfected	infected
**B-cell**	0	15
**CTB**	10	56
**Decidual**	505	633
**Endothelial**	5164	3153
**EVT**	597	149
**LED**	347	104
**Macrophage**	30	244
**npiCTB**	15	3
**STB**	2482	586
**Stromal**	244	811
**T-cell**	107	1145
